# Supplementary material for: Crop pest detection by three-scale convolutional neural network with attention
Source: PLoS One. 2023 Jun 2;18(6):e0276456. doi: 10.1371/journal.pone.0276456 (PMC10237663; doi:10.1371/journal.pone.0276456)
Supplement: Attachment — Submitted filename: Response to Reviewers.docx [file pone.0276456.s001.docx]

### A Summary of Changes

Manuscript Title: Crop Pest Detection by Three-scale Convolutional Neural Network with Attention

**Manuscript ID:**

**Authors:** Shanwen Zhang, Xuqi Wang, Xianfeng Wang, Cong Xu

Dear editors and reviewers,

First of all, we would like to express our most sincere thanks to the Editor-in-Chief, Guest Editors who have provided us so the comments and a chance to improve our work. In the following, let us state the changes to the manuscript and the replies to the reviewers.

**Responses to reviewers' comments:**
Reviewer #1:

1. In abstract, author(s) must show the comparative results in terms of existing method and proposed method.

Response: Thanks for your valuable suggestion.

Experiments are carried out on the image set of common crop pests, and the recognition rate is 93.16%, which is 5.1% and 3.7% higher than ICNN and VGG16, respectively.

2. In Experiments and analysis section author (s) use so many key words (such as Toolkit 9.0, CUDNN V7.0, PYTHON 3.6.4, TENSORFLOW-GPU 1.8.0 etc.), add citation for each key words and also add in reference section.

Response: Thanks for your valuable suggestion. In various papers, the software and hardware conditions of training and testing deep learning models are abnormal. Our software environment, similar to Ref. [14,26], is built as follows: the operating system Win 10 64bit, processor Intel Xeon E5-2643v3@3.40ghz CPU, 64GB memory, NVidia Quadro M4000 GPU, 8GB video memory, CUDA Toolkit 9.0, CUDNN V7.0, Python version 3.6.4, Tensorflow-GPU 1.8.0 framework [14,26].

3. In section 4.2 author(s) says that ' the labeling tool is used..........' at citation for 'Labeling tool in revised manuscript and add in reference section.

Response: Thanks for your finding. We used labelImg tool for labeling the training samples. Its download site is as follows:

abelImg tool (https://github.com/topics/labelimg-tool)

4. Data shows in table-1, must represented using bar graph, so that performance of proposed method can easily compared in terms of precision, Training time and Test time.

Thanks for your valuable suggestion. When comparing the data, the table is not obvious and intuitive, and has been revised as required. The revised bar graph is as follows:

Fig. The detection results of four pest species by six algorithms

We found that the detection results of Precision, Training time and Test time are shown in an image is inappropriate, while shown in following three images is suitable.

(A) Precision	(B) Training time	(C) Test time

Fig. The detection results of four pest species by six algorithms

5. Size of data set 1000 is less what happened if data set increase, what % is used for training and test your proposed method in dataset.

Response: Thanks for your finding. We used five-fold cross-validation scheme to conduct the detecting experiments.

In order to demonstrate the detection performance at different dataset scales, a large number of experiments are conducted and adopted a five-fold cross-validation scheme with different numbers of pest images. Under different number of training samples, the detection results of the proposed method are shown in Fig.8. It can be seen from Fig.8 that the more pest images, the higher the detection accuracy. When the number of training samples is less than 6000, the detection accuracy improves rapidly, when the number of training samples is more than 11,000, the detection accuracy improves stably, while the training samples over 16,000 do not further improve the detection accuracy. The reason is that when the training samples are not enough, the proposed method cannot represent various pest image features, so the detection accuracy is not high. But, when the training samples are large enough, if the training samples are increased, the model TSCNNA training tends to converge, so the detection accuracy will not further increase.

Fig.8 The precision versus the number of the training samples

6. Is the dataset used in the submitted manuscript available for researchers if yes provide link if no why?

Response: Thanks for your finding. We gave our dataset link for free download:

<https://pan.baidu.com/s/1kWEPJDei_fGkPmDAK2OoVA?pwd=p45y>, extraction code: P45Y

7. Important references are missing for CNN, DL and VGG, Introduction of each is required in introduction section. some references are given below which must included in revised manuscript related to CNN, DL and VGG.

Response: Thanks for your finding. We have referenced following 4 references in the revised manuscript related to CNN, DL and VGG.

[12] Barhate D, Pathak S, Dubey A K, et al. Cohort study on recognition of plant species using Deep Learning methods. Journal of Physics: Conference Series. IOP Publishing, 2022, 2273(1): 012006.
[13] Nemade V, Pathak S, Dubey A K. A Systematic Literature Review of Breast Cancer Diagnosis Using Machine Intelligence Techniques. Archives of Computational Methods in Engineering, 2022: 1-30.
[14] Gupta J, Pathak S, Kumar G. Deep Learning (CNN) and Transfer Learning: A Review. Journal of Physics: Conference Series. IOP Publishing, 2022, 2273(1): 012029.
[15] Gupta J, Pathak S, Kumar G. Aquila coyote-tuned deep convolutional neural network for the classification of bare skinned images in websites. International Journal of Machine Learning and Cybernetics, 2022, 13(10): 3239-3254.

8. Is any software implementations available for public use for the proposed algorithms you have proposed, If Yes give the link in revised manuscript, if no why?

Thanks for your valuable suggestion.

Response: Thanks for your finding. We gave our dataset link for free download:

<https://pan.baidu.com/s/1kWEPJDei_fGkPmDAK2OoVA?pwd=p45y>, extraction code: P45Y

9. It is better to divide section-1 into two separate section 1 Introduction 2. Literature Review.

Response: Thanks for your finding. We revised as

1. Introduction

2. Related work.

Reviewer #2:

1. Poor literature survey. Authors can add a separate section of the “Related Works” to investigate the recent published work for proposing the approach.

Thanks for your valuable suggestion. We added a separate section of the “Related Works” to investigate the recent published work for proposing the approach.

2. Author stated that “existing convolutional neural network (CNN) based pest detection methods are not satisfactory for small pest recognition and detection in field”. Give the justification with taking appropriate references. What does mean “small pest recognition and detection in field”.

Thanks for your valuable finding. Appropriate references are as follows:

Small pests often go undetected because much information is lost during the pooling process. In CNN, after a pooling, the output feature map size is reduced by half and the resolution is reduced by half, so the feature of the small pest image may be submerged [23].

[23] Wei [D,](https://www.sciencedirect.com/science/article/abs/pii/S0168169922000539#!) Chen [J,](https://www.sciencedirect.com/science/article/abs/pii/S0168169922000539#!) Luo [T](https://www.sciencedirect.com/science/article/abs/pii/S0168169922000539#!) , et al. Classification of crop pests based on multi-scale feature fusion. [Computers and Electronics in Agriculture](https://www.sciencedirect.com/journal/computers-and-electronics-in-agriculture), 2022, 194: 106736. DoI: 10.3390/insects13060554.

3. Write Fig. 5 instead of Fig. 5AB. It is suggested to proofread completely

Thanks for your valuable finding. We revised Fig. 5 AB as Figs.5 (A) and (B).

4. Contributions can be enhanced, to inform readers the contributions made in advance.

Response: Thanks for your finding. The contributions are summarized as follows.

(1) Multi-scale convolution is used to extract the multi-scale feature for multi-scale pest detection, so that TSCNNA can adapt to pest images of different resolutions.

(2) Attention mechanism is used to enable TSCNNA to focus on crop pests and reduce the impact of background factors, and reduce information loss and accelerate network training.

Finally, thanks again to the reviewers for their comments. Hopefully, we have addressed your concern. If there is any other problem, please let us know.

Sincerely Yours

Xuqi Wang, Shanwen Zhang
